# Interfacial Tailoring of Polyether Sulfone-Modified Silica Mixed Matrix Membranes for CO_2_ Separation

**DOI:** 10.3390/membranes12111129

**Published:** 2022-11-11

**Authors:** Hafiz Abdul Mannan, Alamin Idris, Rizwan Nasir, Hilmi Mukhtar, Danial Qadir, Humbul Suleman, Abdul Basit

**Affiliations:** 1 Chemical Engineering Department, Universiti Teknologi PETRONAS, Bandar Seri Iskandar 32610, Perak, Malaysia; 2 Institute of Polymer and Textile Engineering, University of the Punjab, Lahore 54590, Pakistan; 3 Department of Natural Sciences, Mid Sweden University, 85230 Sundsvall, Sweden; 4 Department of Chemical Engineering, University of Jeddah, Jeddah 23890, Saudi Arabia; 5 School of Computing, Engineering and Digital Technologies, Teesside University, Middlesbrough TS1 3BX, UK; 6 Department of Chemical Engineering, University of Gujrat, Gujrat 50700, Pakistan

**Keywords:** interfacial defects, mixed matrix membrane, modified silica, permeation performance

## Abstract

In this work, in situ polymerization of modified sol-gel silica in a polyether sulfone matrix is presented to control the interfacial defects in organic-inorganic composite membranes. Polyether sulfone polymer and modified silica are used as organic and inorganic components of mixed matrix membranes (MMM). The membranes were prepared with different loadings (2, 4, 6, and 8 wt.%) of modified and unmodified silica. The synthesized membranes were characterized using Field emission electron scanning microscopy, energy dispersive X-ray, Fourier transform infrared spectroscopy, thermogravimetric analyzer, and differential scanning calorimetry. The performance of the membranes was evaluated using a permeation cell set up at a relatively higher-pressure range (5–30 bar). The membranes appear to display ideal morphology with uniform distribution of particles, defect-free structure, and absence of interfacial defects such as voids and particle accumulations. Additionally, the CO_2_/CH_4_ selectivity of the membrane increased with the increase in the modified silica content. Further comparison of the performance indicates that PES/modified silica MMMs show a promising feature of commercially attractive membranes. Therefore, tailoring the interfacial morphology of the membrane results in enhanced properties and improved CO_2_ separation performance.

## 1. Introduction

Developing nanocomposite or mixed matrix membranes are usually preferred over conventional polymeric membranes, owing to their superior membrane property and enhanced gas separation performances. These are generally believed to overcome the permeability–selectivity tradeoff challenge in polymeric membranes. However, the difference in properties among the organic and inorganic constituents of the nanocomposite membranes results in the formation of interfacial defects, which in turn deteriorates one or more properties of the membrane. Interfacial defects in organic–inorganic nanocomposite membranes usually appear in voids in the vicinity of the inorganic filler, pore blockage, poor nanoparticle distribution, formation of particle aggregation, and rigidification of polymer chains around the polymer–particle interface [1]. Such defects are commonly observed in silica-based nanocomposites due to poor compatibility between the organic and inorganic phases. These defects can be minimized or removed by surface modification/functionalization of silica nanoparticles using silane coupling agents while synthesizing silica nanoparticles. The silane coupling agents display a vital bifunctional structure with one terminal capable of reacting with the existing silanol groups on the surface of silica nanoparticles, and the other terminal can interact with the polymer matrix.

Interfacial defects in mixed matrix membranes (MMMs) are the main problem in producing defect-free MMMs on a wide scale. These defects are caused primarily by differences in the thermal and physical characteristics of organic and inorganic phases [2]. Proper polymer and filler selection is critical in the development of high-performance MMMs. Voids develop at the polymer/filler contact due to inadequate adherence. Voids at the interface are commonly referred to as sieve-in-a-cage morphology [3]. The gas passes through these voids because they provide the least resistance to gas flow. Higher permeability and lower/higher selectivity are obtained based on the size of the void. When the size of the void is greater than the permanent gas molecule, the MMM shows higher permeability but no selectivity.

In most cases, low permeability and higher selectivity are obtained when the polymer chains around the polymer particle get rigidified. In some cases, the filler pore is blocked by the solvent or polymer chains often known as pore blockage which causes a decrease in membrane permeability. Particle distribution and aggregation are additional significant difficulties in the development of MMMs, in addition to interfacial defects. The fillers are poorly dispersed, which leads to stress concentration areas that weaken the membrane’s mechanical stability. Additionally, the filler particles aggregate to form no-selective sites. This might lead to a decrease in selectivity and an increase in permeability.

These defects can be overcome by casting the membrane above the glass transition temperature, adding a low molecular weight additive (LMWA), annealing the membrane above *T_g_*, or surface modification of the filler for better adhesion [4]. The compatibility of the polymer and the filler is also a key factor in MMM synthesis. To address this issue, compatibility agents should be utilized. Fabricating defect-free MMMs capable of being turned into membrane modules remains a challenge. These challenges hinder the scale up of MMM to the industrial scale. The common silane coupling agents used for the functionalization/modification of silica nanoparticles are 3-aminopropyl triethoxysilane (APTES), phenyl trimethoxysilane (PTMS), 3-methacryloxypropyle-trimethoxyl silane (MPS), methyl triethoxysilane (MTES), vinyltriethoxysilane (VTES), 3-aminopropyl trimethoxysilane (APTMS), 3-glycidoxypropyl trimethoxysilane (GPTMS), 3-acryloxypropyl trimethoxysilane (APTMOS), etc. Among these agents, those amine-terminated silane coupling agents are most preferred due to their ability to effectively interact with silica nanoparticles and polymers [5].

Yang and Nelson found 3-acryloxypropyl trimethoxysilane (APTMOS) silane coupling agent to be a better choice than 3-acryloxypropyl methydimethoxysilane (APMDMOS) for surface modification of silica nanoparticles [6]. The interfacial defects in polyetherimide/silica MMM were cured by 3-aminopropyltrimethoxysilane (APTMOS) as a coupling agent by Nunes et al. [7]. The coupling agent not only improved the dispersion of silica particles but also reduced the size of silica particles to nanoscale levels. Furthermore, Idris et al. [8] compared the effects of silane agents on the performance of the PC/silica MMMs, where silane agents with varying molecular weights, i.e., 3-aminopropyl(diethoxy)methylsilane, N-(2-aminoethyl)-3-aminopropyltrimethoxysilane, (3-aminopropyl)trimethoxysilane, N-(3-trimethoxysilylpropyl) diethylenetriamine, were investigated. Those silica nanoparticles modified with the lowest molecular weight silane, i.e., (3-aminopropyl) trimethoxysilane with a molecular weight of 179.29 g/mol resulted in monodispersed particles. The corresponding PC/silica membrane property and performance were enhanced compared to the rest silane agents used.

In addition to silane modification, emulsion polymerized mixed matrix (EPMM) has been used to synthesize mixed matrix membranes for gas separation applications. For example, Sadeghi et al. synthesized emulsion polymerized mixed matrix (EPMM) films using poly (2,6-dimethyl-1,4-phenylene oxide) (PPO) in trichloroethylene containing tetraethyl orthosilicate (TEOS). They found that the ideal selectivity is 8% greater than the native PPO for O_2_/N_2_ gas pair [9]. Another study carried out by Bissadi et al. also used the same approach of EPMM to develop nanocomposite membranes using two different co-solvents, ethanol and acetone, and investigated the effect of this technique on transport properties of the resulting PPO-based EPMM membranes [10]. The membranes synthesized by EPMM were found to be more permeable than the neat PPO membrane.

Polyethersulfone (PES) and silica particles are widely used in membrane synthesis for various applications such as oil vapor separation using PES as supporting membrane, and polyether block amide (PEBA) as the separation along with fumed silica [11], flue gas desulphurization using PES/PES-SiO_2_ membrane contractor [12], oily wastewater treatment using direct–contact membrane distillation (DCMD) [13] and water desalination using PES thin film composite membranes [14].

However, no study is reported in the literature on PES polymer as a polymer phase, modified silica nanoparticles synthesized by in situ sol-gel process as the inorganic phase and APTMOS as the coupling agent for MMM fabrication and their testing for CO_2_/CH_4_ separation. PES is a high-performance polymer and possesses a good potential for silica-based MMM fabrication through its in situ sol-gel process. This study differs from Nunes et al., [7] in terms of polymer selection, gas pair separation, and operating conditions. In addition, detailed characterizations and the effect of silica loading on physico-chemical and permeation properties have been reported in the present work. Moreover, this study has been performed to test membranes at a relatively higher-pressure range to explore the potential of these MMMs for natural gas processing applications.

Therefore, in this work, we present the interfacial enhancement of PES/modified silica MMMs for CO_2_ separation, where a silane agent, i.e., (3-aminopropyl) trimethoxysilane, was used to modify the surface of sol-gel silica nanoparticles. PES/modified silica mixed matrix membranes were synthesized via in situ polymerization of the polymer-modified silica sol-gel process. For comparison purposes, PES/unmodified silica mixed matric membranes were prepared in a typical method without using a silane agent. The MMMs with different silica loading (2–8 wt.%) were fabricated via solvent evaporation-dry phase inversion method and then characterized by using Field emission scanning electron microscopy (FESEM), energy dispersive X-ray (EDX), Fourier transform infrared spectroscopy (FTIR), thermogravimetric analyzer (TGA) and differential scanning calorimetry (TGA). The gas separation performance of the MMMs was evaluated using permeation cells at various feed pressure (5–30 bar). The characterization and performance results are analyzed, discussed thoroughly, and presented.

## 2. Materials and Methods

### 2.1. Materials

The base polymer, i.e., Polyether Sulfone (PES), with high molecular weight (50,000 g/mol), was purchased from BASF GmbH, Ludwigshafen, Germany. N-Methyl-2-Pyrrolidone (NMP) with a purity of 99.99% and Hydrochloric acid with 37%wt./wt. the aqueous solution was obtained from Merck. Tetraethyl orthosilicate (TEOS) (98% purity) and (3-aminopropyl) trimethoxysilane (APTMOS) (97% purity) were supplied by Sigma Aldrich. The pure gases CO_2_ and CH_4_ (99.99%) were supplied by Gas Walker Sdn Bhd. Double-deionized water was used for membrane synthesis.

### 2.2. Preparation of Mixed Matrix Membranes (MMMs)

The in situ sol-gel syntheses of silica particles in the PES polymer matrix were employed with some modifications to prepare the MMMs. The procedure is similar to the work reported by Nunes et al. [7], where a known amount of silica precursor, i.e., tetraethyl orthosilicate (TEOS), deionized water, and hydrochloric acid were added to the prepared polymer solution to achieve the desired filler loading. Initially, the PES polymer was dissolved in NMP solvent at 65 °C in a three-neck round bottom flask under constant reflux conditions. Then, a known amount of TEOS was added dropwise to the polymer solution. This was followed by a dropwise addition of a mixture of deionized water and hydrochloric acid to the reaction mixture at 65 °C under rigorous mixing for 3 h to initiate the polymerization reaction of silica. Later, the coupling agent (APTMOS) was added to the dope solution to modify the surface of silica particles. The reaction mixture was then cooled to room temperature to release the air bubbles formed during the mixing process. The dope solution containing PES and modified silica sol was then cast using a casting knife with a predetermined gap of 300 µm on flat, smooth, dry, and dust-free glass plates. The casted membranes were then placed in a natural convection oven at 90 °C for 20 h and then at 130 °C for a subsequent 4 h for densification. A total of 4 mixed matrix membranes with varying modified silica nanoparticle loading were fabricated. The fabricated membranes were labeled as S11, S12, S13, and S14, corresponding to the modified silica loading of 2 wt.%, 4 wt.%, 6 wt.% and 8 wt.%, respectively. Similarly, a typical fabrication method was used to prepare PES/unmodified silica mixed matrix membranes except that the coupling agent (APTMOS) was not added to the dope solution during the solution preparation step. These membranes were labeled S7, S8, S9, and S10. A neat PES membrane (S1) was also prepared as a reference. The composition of the developed membranes and their codes have been tabulated in Table 1.

### 2.3. Characterization Techniques

#### 2.3.1. Morphological Analysis

The morphology of the fabricated MMMs was qualitatively analyzed using a field emission scanning electron microscope (FESEM, ZEISS SUPRATM 55VP). Surface images of the membranes were taken on randomly selected areas. For the cross-sectional images of the membranes, the samples were fractured after being submerged in liquid nitrogen for 0.5 min. The samples were then coated with gold using an Emitech K550X sputter coater. Then, the samples were placed on a sample holder and analyzed at a wide magnification range of 500–50,000× using 15 kV accelerating voltage.

The presence and dispersion of the inorganic fillers in the membrane matrix were investigated using the energy dispersive X-ray (EDX) analysis. The membrane surfaces were subjected to the EDX examination. The EDX spectrum was captured and examined. To track the dispersion of the additives, the elemental mapping of additives was observed.

#### 2.3.2. Spectral Analysis

To identify the functional groups, analyze the interaction between the polymer and the inorganic filler, and the bonding present, Fourier Transform Infrared Spectroscopy (FTIR, Spectrum One/BX) was utilized. FTIR spectrum of the samples was recorded at wavelengths ranging from 400 to 4000 cm^−1^ and analyzed.

#### 2.3.3. Thermal Analysis

The thermal stability of the developed MMMs and the presence of residual solvent were examined using a Thermogravimetric Analyzer (TGA, STA6000, PerkinElmer, Waltham, MA, USA). The samples were first cut into small pieces, and about 5–10 mg weight of the sample was placed in a sample pan. TGA analysis was carried out at temperatures ranging from 30 to 800 °C at a constant heating rate of 10 °C/min under a nitrogen atmosphere.

The glass transition temperature (*T_g_*) of the membranes was characterized by using Differential Scanning Calorimetry (DSC, Q2000 TA). Samples were cut into small pieces with an average weight of 5–10 mg. The thermal scans were performed from 50 to 250 °C under nitrogen flow with a heating rate of 20 °C/min, a cooling scan of 250–0 °C and a second heating scan performed at the typical rate, i.e., 20 °C/min.

#### 2.3.4. X-ray Diffraction (XRD)

X-ray diffractometer (Model: X’Pert3 Powder, PANayltical, Malvern, UK) was used for XRD analysis of membrane samples. The analysis was performed at room temperature at 2 theta range from 2° ≥ 2θ ≥ 40°.

### 2.4. Performance Analysis

The performance of flat sheet membranes was evaluated using a constant pressure variable volume gas permeation unit. The membrane cell is a dead-end membrane test module with an effective area of 19.54 cm^2^. Prior to the experiments, the unit was vacuumed for 30 minutes to evacuate any residual gases. The membranes were tested with the feed gas at a relatively higher-pressure range at different pressures, i.e., 5, 10, 15, 20, 25, and 30 bar at 25 °C. For each membrane sample, at least 4 trials of permeation experiments were performed. The experimental error is within ±5% of the reported values in the permeation experiments. The permeate flow rates were recorded using a digital bubble flow meter. The permeability of CO_2_ gas was calculated by the following equation [10]:

(1) $$P_{CO_{2}}=\frac{J_{CO_{2}}l}{\Delta p_{CO_{2}}}$$

where *J* is the flux (*Q_stp_/A*) of CO_2_ gas across the membrane, *l* is membrane thickness, and ∆*p* is the pressure difference across the membrane. *Q_stp_* refers to the volumetric flow rate of permeating gas corrected to standard temperature and pressure conditions and *A* is membrane area. The average thickness of the membranes is in the range of 40–59 µm.

Similarly, the permeability of CH_4_ gas can be obtained from the above equation. The permeability was reported in the unit of barrer. The ideal selectivity of the membrane was calculated as the ratio of permeability of CO_2_ to CH_4_ gas.

## 3. Results and Discussion

### 3.1. Morphological Analysis

**Figure 1:**
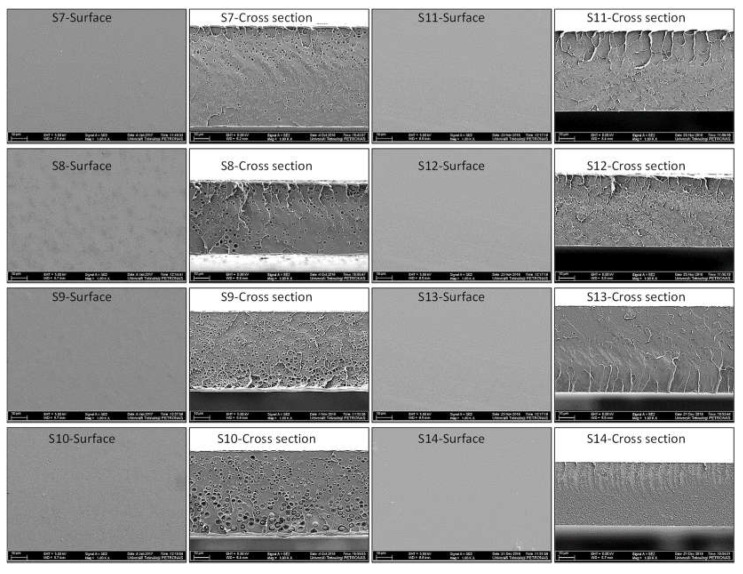
FESEM images of MMMs at 1000×.

The surface and cross-section morphology of the synthesized MMMs at various silica loadings, i.e., 2, 4, 6, and 8 wt.%, is shown in Figure 1. Although the distribution of the silica particles is fairly placed, the particle sizes of the silica particle appear to be large in PES/unmodified silica MMMs, with no agglomeration of silica particles observed on the MMMs, which suggests the uniform dispersion of the silica particles [15]. These observations show that silica particles have been embedded effectively into the polymer matrix via sol-gel process, and a fairly uniform dispersion is achieved.

**Figure 2:**
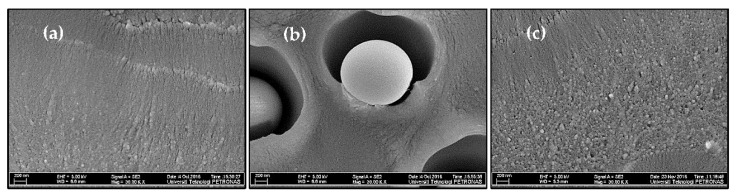
Comparison of the morphology of (**a**) Pure PES membrane (S1), (**b**) PES/unmodified silica MMM (S8), and (**c**) PES/modified silica MMMs (S11) at 30,000×.

According to Figure 1, it can be observed that the membranes polymer chain packing is disrupted by adding silica particles in the PES matrix which can be observed by comparing pure PES membrane (Figure S1, in Supplementary Materials) and MMMs. For pure PES membrane, no void formation was observed in the cross-section due to the absence of the particles as shown in Figure S1. However, for MMMs with various unmodified silica loadings, void formation around the silica particles is observed, which is more prominent at higher silica loadings (6 and 8%). This morphology is a typical example of sieve-in-a-cage morphology where filler particles are surrounded by interfacial voids in the membrane, as shown in Figure 2b [3,16]. These interfacial voids are present because no interaction between silica and PES matrix is present, resulting in poor adhesion between the inorganic and polymer phases [17]. Additionally, these defects are observed in MMM because polymer and inorganic have different phase properties and thermal behaviors. Upon solvent evaporation during the membrane synthesis process, the difference in these phase properties and thermal expansions results in non-uniform distribution of stresses along polymer and inorganic phases, which causes interfacial defects or voids [18]. Moore and Koros also observed sieve-in-a-cage morphology for polysulfone (Udel^®^)/zeolite 4A polymer filler system due to poor adhesion between the polymer and the filler [19]. Similarly, Shen and Lua also observed the interfacial defects and voids in the PI/unmodified silica nanoparticles system [20].

To avoid interfacial defects in the form of interfacial voids and the formation of particle agglomerates within the membrane morphology, the silica particles were modified with (3-aminopropyl) trimethoxysilane (APTMOS) coupling agent. Figure 1 also shows the surface and cross-section morphology of the MMMs based on modified silica nanoparticles. Surface images reveal a homogenous dense surface with no pinhole defects, voids, or particle agglomeration. This observation is consistent for all MMMs regardless of the silica loading. Thus, the effective embedding of silica nanoparticles in the PES matrix is achieved after surface modification of nanoparticles via the in situ sol-gel process.

The cross-sectional images of PES/modified silica MMMs reveal the changes induced in the PES matrix after surface modification of silica nanoparticles with an amine coupling agent. Similar to the pure PES membrane, PES/modified silica MMMs are dense, symmetric, and homogenous. The incorporation of silica particles has interrupted polymer chain packing and created a typical morphology of MMMs where nanoparticles are uniformly distributed in the polymer matrix.

In the synthesized PES/modified silica MMMs, silica nanoparticles are embedded in such a way that they are not easily distinguishable as a different phase in the polymer matrix as can be seen in Figure 2 where the morphology of pure PES, PES/unmodified silica MMMs and PES/modified silica MMMs is compared. There is no difference between PES membrane and PES/modified silica MMMs in terms of homogeneity. The white spherical shapes in Figure 2c are silica domains surrounded by and embedded in the polymer matrix. Similar results are reported for PEI/modified silica MMMs [7].

This observation suggests the improved compatibility and strong interaction between modified silica nanoparticles and polymer matrix. The hydrophobicity of the surface of the inorganic particles is enhanced by a silane coupling agent resulting in the entanglement of filler with polymer chains [20]. Apparently, no voids or visible defects at the polymer/filler interface are observed in PES/modified silica MMMs, unlike PES/unmodified silica MMMs. The silica surface modification enabled the interaction between the polymer and the filler.

Careful inspection of Figure 2 shows that a few voids in the nano-range are still observable in PES/modified silica MMMs, indicating the presence of additional void volume besides fractional free volume. Interestingly, the particle size is reduced to nanoscale after amine modification of silica filler due to probable hydrogen bonding and affinity between the inorganic and polymer phases. Idris et al. [18] reported polycarbonate/modified silica nanocomposite membranes prepared via the post-modification method. The morphology of the membranes showed interfacial defects and rigidified polymer layers around the silica nanoparticles. However, with the in situ sol-gel functionalization method adopted in the current study, the defects were reduced to a great extent after silane modification of silica nanoparticles for PES/silica nanoparticles systems. More interestingly, uniform dispersion and the adhesion between the inorganic and polymer phases have been improved. These observations suggest that silica nanoparticles have been embedded effectively into the polymer matrix via the in situ sol-gel process. Thus, the membranes approaching the ideal morphology have been synthesized at maximum silica loading (8%), unlike unmodified silica particles, where defects were observed even at the lowest loading of 2%.

### 3.2. EDX Mapping Analysis

**Figure 3:**
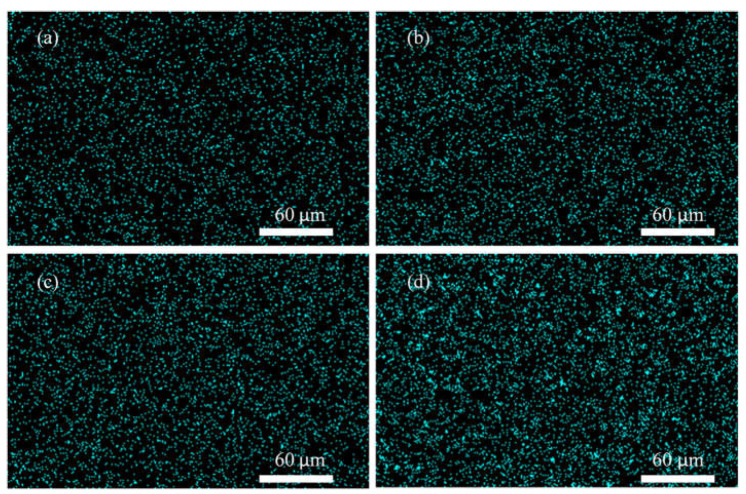
Mapping of silicon in PES/unmodified silica MMMs (**a**) S7 (**b**) S8 (**c**) S9 and (**d**) S10.

**Figure 4:**
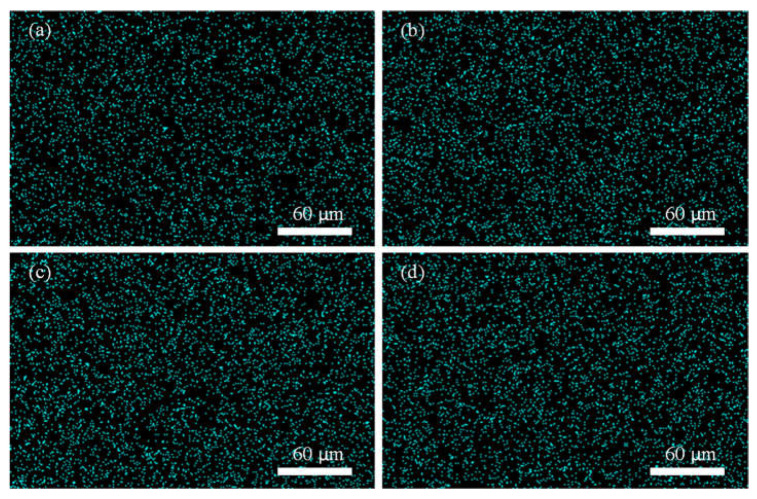
Mapping of silicon in PES/modified silica MMMs (**a**) S11 (**b**) S12 (**c**) S13 and (**d**) S14.

The synthesized MMMs were further characterized for the distribution of silica nanoparticles. Figure 3 and Figure 4 show the EXD mapping of silicon element in PES/unmodified silica and PES/modified silica MMMs. The silica is well distributed and dispersed in the membranes at lower loading. However, at higher silica loading (8%) in PES/unmodified silica MMMs, a few agglomerates are seen in Figure 3d. No agglomeration is observed in Figure 4 suggesting a homogenous and uniform distribution of silica for MMMs after the surface modification of silica nanoparticles. Thus, silane modification of silica nanoparticles has improved the dispersion of the filler in MMMs.

### 3.3. Structural Analysis

**Figure 5:**
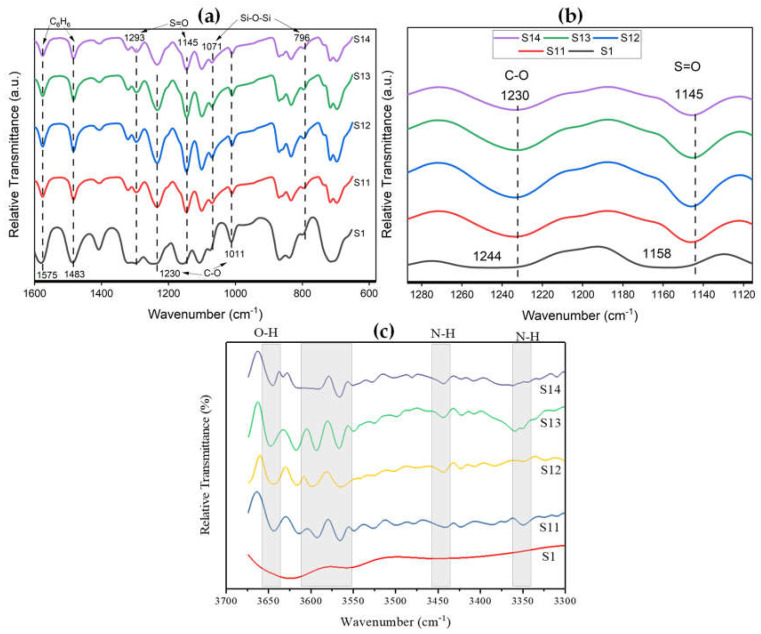
FTIR spectra of PES/modified silica MMMs (**a**) in fingerprint region (**b**) detailed spectra from 1120–1300 cm^−1^ (**c**) detailed spectra from 3300–3700 cm^−1^.

Detailed FTIR spectra of the synthesized MMMs is shown in Figure 5. The characteristic bands of silica and PES matrix are observed and labeled accordingly in Figure 5a. There is no difference in the spectra of MMMs in the fingerprint region as can be seen Figure 5a. This implies that PES is chemically unaffected by incorporating modified silica nanoparticles. However, a remarkable spectral shift in ether linkage (C–O) and sulfone group (S=O) is observed after the incorporation of modified silica nanoparticles in the PES matrix, as shown in Figure 5b where the spectra of ether linkage and sulfone groups are enlarged. The ether link band shifted from 1244 cm^−1^ for pure PES membrane to 1230 cm^−1^ for MMMs. Similarly, the sulfone peak shifted from 1158 cm^−1^ for pure PES membrane to 1145 cm^−1^ for MMMs. These spectral shifts towards lower wavelength indicate the presence of strong interaction such as hydrogen bonding between the two phases at the molecular level [21,22,23,24]. The functionalization of silica nanoparticles with silane coupling agent is confirmed by the presence of two amine bands (N–H) at 3500–3300 cm^−1^ since the silane coupling agent contains (N–H) group in its structure, as shown in Figure 5c [20,25]. In addition, silanol groups (Si–OH) are also observed at 3640 cm^−1^ due to their natural occurrence on silica surfaces [26,27]. The presence of (N–H) and (Si–OH) groups in modified silica nanoparticles interacted with the PES matrix, causing a spectral shift in sulfone and ether link bands.

The spectral shifts in sulfone and ether links are due to a decrease in electron density shared between S and O, and C and O, respectively, because of interaction between O in sulfone and ether groups and H in the amine group. The non-condensed O-H groups from silica and N–H groups from the silane coupling agent provided the connections between the PES and silica phases. Similar manifestation of spectral shifts in PC/modified silica and PSF/PI blend/modified silica was also reported in the literature [18,28].

### 3.4. Thermal Stability Analysis

**Figure 6:**
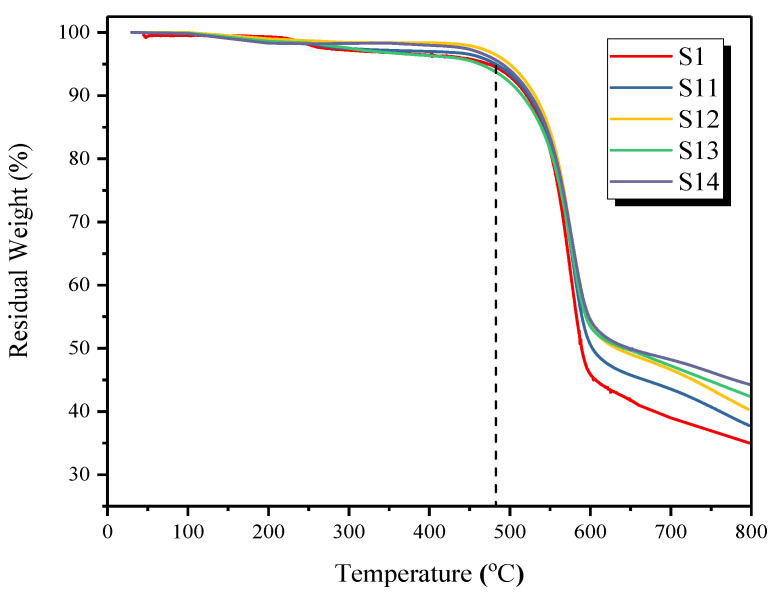
TGA thermograms of pure PES membrane and PES/modified silica MMMs.

Figure 6 displays the TGA thermograms of the pure PES membrane and the synthesized MMMs with modified silica nanoparticles. The thermogram of all the membranes shows that there is no significant residual solvent or moisture in the MMMs, indicating solvent’s complete evaporation during the membrane drying process. The degradation onset temperature of MMMs is slightly higher than PES membrane (473.5 °C). For example, degradation onset temperature for S11, S12, S13, and S14 membranes containing 2%, 4%, 6%, and 8% of modified silica is found to be 482.1 °C, 499.37 °C, 503.13 °C and 507.21 °C, respectively. While this degradation onset temperature for the PES membrane was found to be 473.05 °C. Thus, it is obvious that the incorporation of modified silica nanoparticles has enhanced the thermal stability of the membranes and delayed the degradation process [28]. Moreover, in the thermal decomposition range of 470–600 °C, more residual weight is observed for MMMs, indicating the incorporation of silica particles in the PES matrix. Thus, at 600 °C, S11, S12, S13, and S14 membranes containing 2%, 4%, 6% and 8% of modified silica has shown 50.60%, 52.03%, 53.46% and 54.51% residual weight, respectively. Similar improvements in the thermal stability of MMMs by incorporating fillers are reported in the literature [29].

The final residual yield for developed MMMs is also in an increasing trend proportional to the amounts of silica loading. PES/modified silica MMMs containing 2%, 4%, 6% and 8% of modified silica have shown 37.79%, 40.35%, 42.42%, and 44.26% residual weights, respectively, as can be observed in Figure 6. This observation indicates the successful incorporation of silica and appreciable dispersion in the PES matrix.

### 3.5. DSC Analysis

The DSC results of MMMs encompassing various loadings of modified silica particles are shown in Table 2. For all MMMs, a single distinct *T_g_* was observed, which shows the presence of a single polymer phase [30] and the compatibility of modified silica nanoparticles in the PES matrix at the molecular level [31]. There is no significant difference between the *T_g_* of pure PES membrane and the *T_g_* of MMMs with only slightly higher *T_g_* values.

### 3.6. Gas Permeation Performance

The performance of the fabricated MMMs was measured using permeation cell for single gas viz., CO_2_ and CH_4_. The effects of modified silica loading on the performance of the membranes were evaluated at 5 bar gas pressure and room temperature. To investigate the effects of feed pressure on the performance of the membranes, the measurement has been performed at various feed pressures ranging from 5 to 30 bars.

#### 3.6.1. Effect of Modified Silica Loading

**Figure 7:**
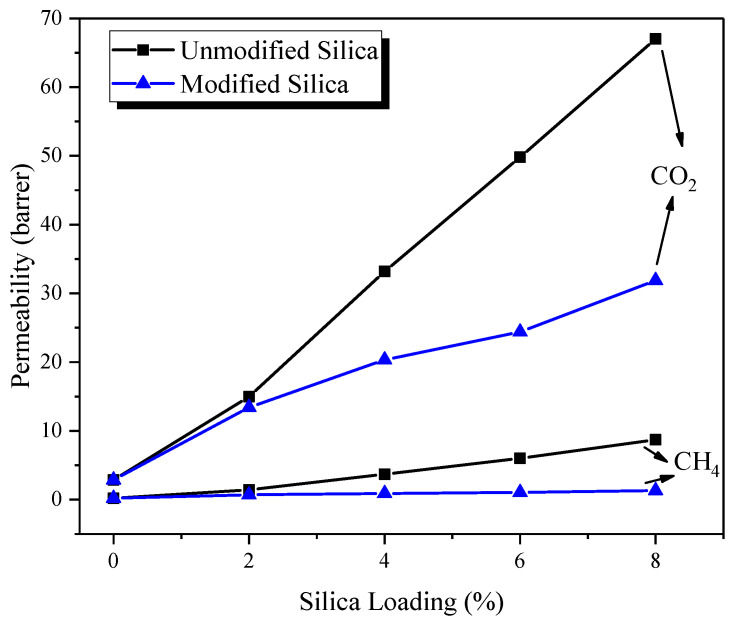
Comparison of permeability in PES/unmodified silica MMM and PES/modified silica MMMs.

Figure 7 shows CO_2_ and CH_4_ gas permeability in MMMs with various silica loading. The permeability of CO_2_ and CH_4_ gases increased with increasing silica loading in MMMs. For instance, at 2% loading of modified silica, CO_2_ permeability is 13.42 barrer, at 4%, CO_2_ permeability is 20.33 barrer, while at 6 and 8% loadings, the permeability is 24.39 and 31.88 barrer, respectively shown in Table 3. Similarly, when the modified silica loading increased from 0–8%, the corresponding CH_4_ permeability increased from 0.20 to 1.32 barrer, respectively. However, unlike unmodified silica-filled MMMs, the rise in the permeability of modified silica-filled MMMs is not sharp for CO_2_ and CH_4_ gases. This behavior is because the interfacial defects and voids were more dominant in PES/silica MMMs. In PES/modified silica MMMs these defects were eliminated largely due to good adhesion between the filler and the polymer. Thus, PES/modified silica MMMs controlled the non-selective bypass of fast and slow-moving gases, i.e., CO_2_ and CH_4,_ respectively, through the membrane matrix, thereby reducing the permeability of both gases compared to unmodified silica filled MMMs. Since silica is a non-porous filler, no passage is available for gas transport through the filler. Most of the permeating gas is transported through the polymer matrix, i.e., PES which provides a very selective but highly resistive path for gas transport. On the other hand, only a small fraction of gas can pass through the polymer-filler interface and filler agglomerates in accordance with Knudson diffusion [32]. This behavior is widely reported in the literature for silica-filled polymer MMM systems [18,33,34,35,36].

Nevertheless, the permeability of CO_2_ is greater than CH_4_ gas for all loadings of silica. This is due to the smaller CO_2_ molecules penetrating faster than bigger CH_4_ molecules in the membrane matrix. Thus, the sieving ability of the membrane is retained at various silica loadings. Another factor contributing to the higher permeability of CO_2_ gas compared to pure PES membrane is the presence of functional groups at the filler surface, such as hydroxyl groups and amine groups resulting from silane modification of filler [18]. These groups tend to interact with polar gases such as CO_2_ instead of non-polar gas, i.e., CH_4_. Thus, a slightly higher solubility is expected in amine-modified silica-filled MMMs compared to pure PES membrane. Therefore, higher CO_2_ permeability is observed with the increase in silica loading. In the case of CH_4_, the permeability increase is attributed to interfacial volumes at the polymer-filler interface. The third factor, besides the interfacial morphology and the presence of amine functional groups, is the disruption of the polymer chain packing and creation of excess free volume in the membrane structure due to the incorporation of filler, as it was reported for unmodified silica-filled MMMs [37]. This observation is supported by XRD pattern of the synthesized MMMs shown in Figure S2 where 2θ is slightly shifted from 18.10° to 17.42° showing an increase in the amorphous structure of the PES matrix [38]. This free volume provides an additional pathway for gas diffusion in inorganic-filled MMMs. The change in the solubility coefficient of MMMs is negligible at lower filler loadings [20]. Therefore, the enhancement in permeability is mainly due to a rise in the diffusion coefficient induced by the creation of excess free volume in the membrane matrix.

Table 3 shows the change in FFV in MMMs after the incorporation of various loadings of modified silica in MMMs. The FFV data have been calculated by the buoyancy method [39,40] and details have been described elsewhere [41]. A gradual increase in FFV of MMMs is observed as the silica loading is increased from 2 to 8%. This might be due to strong interaction and acceptable compatibility between the filler and polymer phases in modified silica-filled MMMs. Shen and Lua [20] also observed a similar FFV behavior for unmodified and modified filled PI-based MMMs.

**Figure 8:**
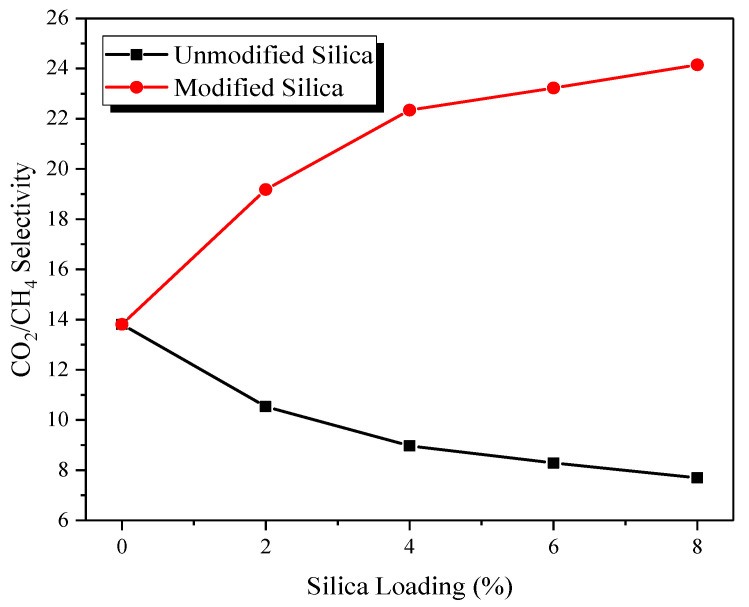
Effect of modified silica loading on ideal selectivity of MMMs.

Figure 8 shows the selectivity of MMMs at various loadings of modified silica nanoparticles, tested at 5 bar and room temperature. The CO_2_/CH_4_ selectivity of MMMs demonstrates an increasing trend with the increase in silica loading. For example, the selectivity of pure PES membrane is 13.80, whereas, at 2, 4, 6, 8% modified silica loading, the selectivity of MMMs is 19.18, 22.34, 23.22, and 24.15, respectively. The increase in selectivity of MMMs compared with pure PES membrane is calculated as 38.98, 61.88, 68.26 and 75% when modified silica loading increases from 2 to 8%, respectively.

This increase in the selectivity of MMMs is due to the absence of interfacial voids and defects in the membrane structure and better polymer-filler interfacial properties. In MMMs, the composite membranes maintained their size-sieving ability due to a better polymer-filler interface. Moreover, the increase in FFV and affinity between CO_2_, and OH and NH groups on silica nanoparticles leads to a higher CO_2_ permeability than CH_4_ permeability. The combined effects of enhanced CO_2_ diffusivity and solubility in MMMs impart high selectivity to MMMs compared with pure PES and MMMs. A similar trend has been reported for modified silica particles in PI/SiO_2_ MMMs [20]. Recently, Idris et al., [18] also observed an increase in the selectivity of PC/modified silica MMMs.

#### 3.6.2. Effect of Feed Pressure

**Figure 9:**
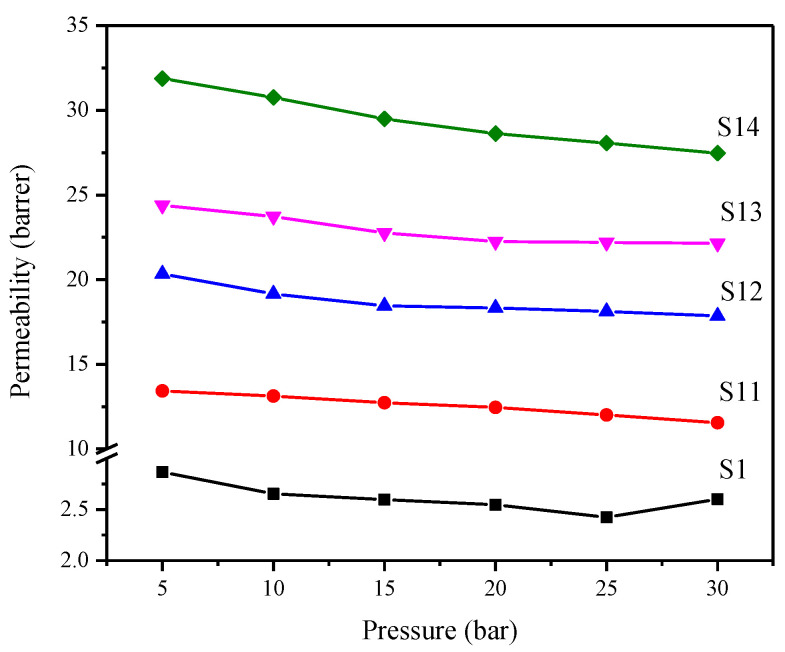
Effect of feed pressure on CO_2_ permeability of PES/modified silica MMMs.

PES/modified silica MMMs were tested at elevated feed pressure (5–30 bar) to study the effect of feed pressure on CO_2_ permeation. Figure 9 shows the effect of feed pressure on CO_2_ permeability of MMMs, and the results are compared with pure PES membrane. The pure PES membrane has a decreasing trend in permeability with the increase in feed pressure, a known characteristic of glassy polymers in accordance with the dual-mode sorption model. A similar trend has been observed for PES/modified silica MMMs which shows the glassy nature of MMMs [42]. The decrease in permeability of MMMs with increasing feed pressure is attributed to a decrease in the solubility coefficient for glassy polymers at high pressures [43].

For MMMs with modified silica nanoparticles, the maximum permeability is recorded at the lowest pressure, i.e., 5 bar and the minimum permeability is observed at the highest tested pressure, i.e., 30 bar. However, the trend is different for pure PES membrane and MMMs. In pure PES membrane, the plasticization phenomenon is observed after 25 bar feed pressure. The reported plasticization pressure of PES is 28 bar for CO_2_ gas [44]. As the pressure is further raised, more CO_2_ gas is sorbed into the polymer matrix, causing polymer chains to relax and increase chain mobility. The combined effect of increased chain mobility and swelling of the polymer matrix causes an increase in the diffusion of gas molecules by lowering the activation energy required for permeation. Because of this phenomenon, the ability of membranes to discriminate between gases based on molecular size is significantly reduced, lowering the selectivity [43,45].

Contrarily, in MMMs, no such trend is observed even at 30 bar, suggesting that silica nanoparticles’ incorporation has imparted plasticization resistance to PES-based MMMs. This indicates the suppression of plasticization phenomena in the PES matrix after incorporating modified silica nanoparticles. Nanoparticles are reported to suppress the plasticization phenomena in MMMs by restricting the chain mobility in nanoparticles filled membranes compared to unfilled polymer membranes [43,45]. Thus, incorporating modified silica nanoparticles improved the separation performance and suppressed the plasticization phenomena in PES-based MMMs.

**Figure 10:**
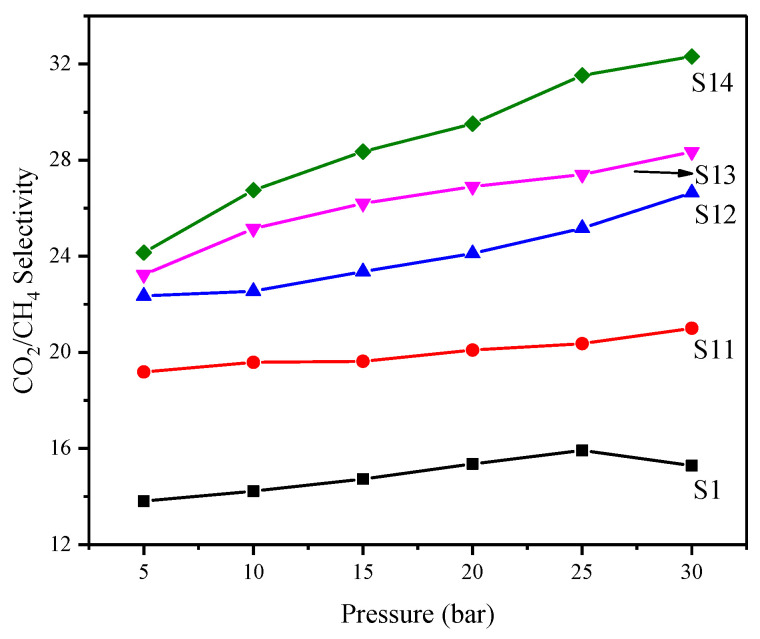
Effect of feed pressure on the selectivity of PES/modified silica MMMs.

The ideal selectivity of MMMs is shown in Figure 10 in the pressure range of 5–30 bar. The ideal selectivity of the synthesized MMMs increases with feed pressure, a reported trend in glassy polymers. Due to a reduction in the permeability of MMMs at high pressures resulting from compaction of the matrix, the size sieving ability of the MMMs is improved, which imparts selectivity to MMMs. All MMMs displayed maximum selectivities at the maximum operating pressure of 30 bar. The maximum selectivity of 32.31 is observed at 30 bar for MMM with maximum silica loading, i.e., 8%. Thus, MMMs were able to maintain permeability as well as selectivity within the study’s pressure range. These results indicate that PES/modified silica filled MMMs have a great potential in CO_2_/CH_4_ separation at elevated pressures.

#### 3.6.3. Comparison of MMMs on Robeson Upper Bound Line

**Figure 11:**
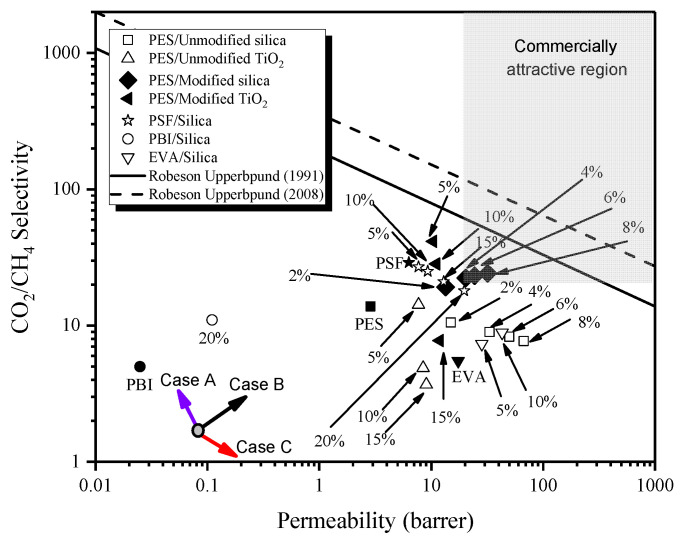
Comparison of MMMs on Robeson upper bound.

Figure 11 shows the performance of the synthesized MMMs on the Robeson upper bound curve. For comparison, PES/amine-modified TiO_2_ MMMs were also included [46]. The permeation data for PSF/SiO_2_ [47], PES/unmodified SiO_2_ (from this work), PES/unmodified TiO_2_ [46], PBI/SiO_2_ [26], and EVA/SiO_2_ [21] MMM systems is also included in the comparison.

PES membrane displays a reasonable separation performance compared to PBI and EVA polymers. The permeability-selectivity tradeoff in MMMs usually shows three types of behavior [3]; case A, case B and case C. Case A is related to matrix rigidification or partial pore blockage of particles pore by polymer chain in MMMs. Thus, non-ideal morphology causes a decrease in permeability with an increase in selectivity. Case B signifies the desired behavior, which is a concurrent increase in selectivity and permeability due to the compatibility of the material. PES/modified silica MMMs at all loadings, PES/modified TiO_2_ MMM at lower loading (5%), EVA/SiO_2_ MMMs, and PBI/SiO_2_ MMM display this kind of behavior. Case C is a typical example of MMM having interfacial defects and non-selective voids due to incompatibility between the polymer and the filler. Therefore, increased permeability with decreased selectivity is expected to be observed. Unmodified MMM systems shown in Figure 11, such as PSF/SiO_2_, PES/unmodified SiO_2_, and PES/unmodified TiO_2_ follow this behavior. PES/modified TiO_2_ MMM at higher loadings (10 and 15%) also exhibit this kind of behavior due to the agglomeration of TiO_2_ nanoparticles. The performance of all MMMs shown in Figure 11 is limited by the upper bound curve. However, the separation performance of PES/modified silica MMMs is approaching the upper bound limit and shows a good combination of permeability and selectivity.

The increase in the permeability of MMMs is attributed to the creation of excess free volume in the membrane by disrupting the polymer chain packing and attachment of active functional groups on the filler surface, which have an affinity with target gas, i.e., CO_2_. The improvement in the selectivity of MMMs is attributed to the elimination of interfacial defects. Moreover, PES/modified silica MMMs fall in the commercially attractive region, as displayed by the grey area in the upper bound curve [48]. Therefore, PES/modified silica MMMs have emerged as a potential candidate for application in natural gas processing applications.

## 4. Conclusions

In the present work, interfacial tailoring of Polyether Sulfone/modified silica mixed matrix membranes was carried out by in situ sol-gel polymerization. From morphological analysis, it was observed that interfacial voids and defects were present in PES/unmodified silica mixed matrix membranes (MMMs) due to poor adhesion between PES and unmodified silica particles. The interfacial defects were successfully cured by surface modification of silica filler by amine terminated coupling agent (APTMOS) in MMMs. Thus, MMMs exhibit ideal morphology with uniform distribution of particles, defect-free structure, and absence of interfacial defects such as voids and particle agglomerations of particles. FTIR spectra observed physical interaction between PES and modified silica, which improved the membranes’ morphology. In MMMs, an increase in permeability and selectivity was observed as compared to pure PES membrane, which is attributed to the creation of excess free volume in the membrane by the disruption of polymer chain packing and attachment of active functional groups on the filler surface. The synthesized MMMs maintained their permeation performance at a relatively higher-pressure range of 5–30 bar, and no plasticization behaviour was observed in MMMs within the studies pressure range. Thus, the incorporation of modified silica nanoparticles in PES matrix restricted the chain mobility and suppressed the plasticization phenomena in PES glassy polymer. The separation performance of PES/modified silica MMMs falls in the commercially attractive region on Robeson upper bound limit. These interfacially tailored MMMs have emerged as potential candidates for use in CO_2_ capture and natural gas processing applications.

## Figures and Tables

**Table 1:** Composition of pure PES membrane and PES/unmodified and PES/modified silica MMMs and their coding at various silica loadings.

Description	Membrane ID	Silica Composition (wt.%)
Pure PES membrane	S1	0
PES/unmodified silica MMMs	S7	2
	S8	4
	S9	6
	S10	8
PES/modified silica MMMs	S11	2
	S12	4
	S13	6
	S14	8

**Table 2:** *T_g_* of pure PES membrane and PES/modified silica MMMs at various silica loadings.

Membrane	Description	*T_g_* (°C)
S1	Pure PES membrane	204.6
S11	Silica = 2 wt.%	205.3
S12	Silica = 4 wt.%	205.9
S13	Silica = 6 wt.%	206.3
S14	Silica = 8 wt.%	206.6

**Table 3:** Effect of modified silica loading on FFV of MMMs and gas permeation.

Silica Loading (%)	FFV	Percentage Increase in FFV (%)	CO_2_ Permeability (Barrer)	CH_4_ Permeability (Barrer)
0	0.141	0	2.86	0.20
2	0.163	15.60	13.42	0.70
4	0.170	20.57	20.33	0.91
6	0.173	22.70	24.39	1.05
8	0.179	26.95	31.88	1.32

## Data Availability

Not applicable.

## Supplementary Materials

The following supporting information can be downloaded at: https://www.mdpi.com/article/10.3390/membranes12111129/s1. Figure S1. FESEM images of pure PES membrane (a) surface (b) cross section at 1000×. Figure S2. XRD pattern of the synthesized MMMs.
