# Supplementary material for: Interfacial Tailoring of Polyether Sulfone-Modified Silica Mixed Matrix Membranes for CO2 Separation
Source: Membranes (Basel). 2022 Nov 11;12(11):1129. doi: 10.3390/membranes12111129 (PMC9698322; doi:10.3390/membranes12111129)
Supplement: Supplementary file 1 [file membranes-12-01129-s001.zip › membranes-1953444-supplementary.pdf]

# Supplementary Information

## Interfacial Tailoring of Polyether Sulfone-Modified Silica Mixed Matrix Membranes for $CO_2$ Separation

**Hafiz Abdul Mannan [1,2], Alamin Idris [3], Rizwan Nasir [4], Hilmi Mukhtar [1], Danial Qadir [5], Humbul Suleman [5,*], Abdul Basit [6]**

[1] Chemical Engineering Department, Universiti Teknologi PETRONAS, Bandar Seri Iskandar 32610, Perak, Malaysia

[2] Institute of Polymer and Textile Engineering, University of the Punjab, Lahore 54590, Pakistan

[3] Department of Natural Sciences, Mid Sweden University, Sundsvall 85230, Sweden

[4] Department of Chemical Engineering, University of Jeddah, Jeddah 23890, Saudi Arabia

[5] School of Computing, Engineering and Digital Technologies, Teesside University, Middlesbrough TS1 3BX, UK

[6] Department of Chemical Engineering, University of Gujrat, Gujrat 50700, Pakistan

* Correspondence h.suleman@tees.ac.uk

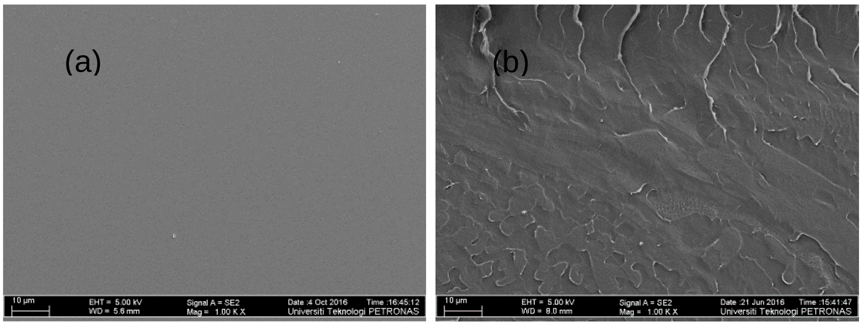


**Figure S1.** FESEM images of pure PES membrane (a) surface (b) cross section at 1000x.

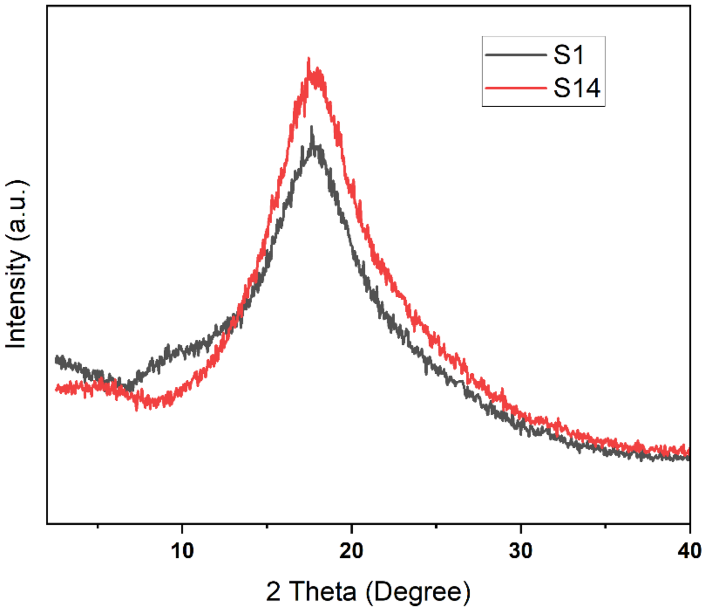

**Figure S2.** XRD pattern of the synthesized MMMs.
